# Application of machine learning prediction models for in-hospital mortality in diabetic sepsis patients: a study based on the MIMIC IV database

**DOI:** 10.1186/s12911-026-03580-4

**Published:** 2026-10-22

**Authors:** Zongxin Shen, Haijiao Zhang, Yu Wang

**Affiliations:** Department of Anesthesiology, Shanghai Jing’an District Zhabei Central Hospital, Shanghai, People’s Republic of China

**Keywords:** Sepsis, Diabetes mellitus, Intensive care unit, Machine learning, Mortality prediction

## Abstract

**Background:**

Sepsis complicated by diabetes mellitus represents a significant clinical challenge in intensive care units (ICUs), associated with substantial morbidity and mortality. Accurate prognostication of in-hospital mortality in this complex patient population is crucial for optimizing clinical decision making, resource allocation, and patient management strategies.

**Methods:**

We conducted a retrospective cohort study utilizing data from the Medical Information Mart for Intensive Care (MIMIC-IV) database. The study focused on adult ICU patients with concurrent diagnoses of sepsis and diabetes mellitus. Six machine learning algorithms were evaluated for their predictive performance: Logistic Regression (LR), K-Nearest Neighbors (KNN), Random Forest (RF), Gradient Boosting Decision Tree (GBDT), XGBoost and LightGBM. Additionally, we assessed the prognostic capability of the Sequential Organ Failure Assessment (SOFA) score. Model discrimination was quantified using the area under the receiver operating characteristic curve (AUC). To enhance model interpretability, we employed SHAP (SHapley Additive exPlanations) values to elucidate the relative importance of predictor variables.

**Results:**

The study cohort comprised 7716 patients, with 1357 (17.6%) experiencing in-hospital mortality. Among the machine learning models evaluated, GBDT exhibited superior predictive performance achieving an AUC of 0.792 (95% CI: 0.769–0.814). This performance surpassed that of other machine learning algorithms and the conventional SOFA score. SHAP analysis provided valuable insights into the hierarchical importance of various clinical and demographic features contributing to the GBDT model’s predictions.

**Conclusion:**

Our findings demonstrate the potential of machine learning approaches in accurately predicting in-hospital mortality among ICU patients with sepsis and diabetes mellitus. These advanced analytical techniques outperform traditional scoring systems such as SOFA. The integration of SHAP values enhances model interpretability, potentially facilitating the adoption of these predictive tools in clinical practice. Further multi-center validation studies and prospective trials are warranted to confirm the generalizability of these results and to explore the clinical impact of implementing such models in diverse healthcare settings.

**Supplementary Information:**

The online version contains supplementary material available at https://doi.org/10.1186/s12911-026-03580-4.

## Introduction

Sepsis complicated by diabetes mellitus represents a significant clinical challenge in intensive care units (ICUs), associated with substantial morbidity and mortality [1]. This complex interplay between two major health concerns has become increasingly prevalent, necessitating a deeper understanding of their combined impact on patient outcomes. Recent global estimates suggest that sepsis affects approximately 48.9 million individuals annually, resulting in 11 million deaths [2]. Among sepsis patients, the prevalence of diabetes mellitus ranges from 17% to 30%, highlighting the frequent co-occurrence of these conditions and underscoring the importance of addressing this specific patient population [3, 4].

The relationship between sepsis and diabetes is intricate and multifaceted, with diabetes known to alter immune function and potentially modify the host response to infection and sepsis [5]. This altered physiological state can lead to unique challenges in diagnosis, treatment, and prognostication. The dysregulated glucose metabolism characteristic of diabetes may exacerbate the metabolic derangements seen in sepsis, potentially leading to more severe organ dysfunction. Conversely, the chronic inflammatory state associated with diabetes might paradoxically prepare the immune system for the acute insult of sepsis. However, the impact of pre-existing diabetes on sepsis outcomes remains a subject of debate within the medical community. The literature presents conflicting findings, with some studies reporting increased mortality in septic patients with diabetes [6], while others have found no significant difference or even suggested a potential protective effect [7, 8]. These contradictory results highlight the need for more sophisticated analytical approaches to unravel the complex interactions between these two conditions and their impact on patient outcomes.

Accurate prognostication of in-hospital mortality in sepsis patients with diabetes is crucial for optimizing clinical decision-making, resource allocation, and patient management strategies. The ability to identify high-risk patients early in their clinical course could facilitate more aggressive interventions, closer monitoring, or the implementation of tailored treatment protocols. Traditional scoring systems, such as the Sequential Organ Failure Assessment (SOFA) score, have been widely used for risk stratification in sepsis [9]. These tools often rely on a limited set of variables and may not fully capture the complex interactions between sepsis and diabetes. In recent years, machine learning approaches have shown promise in improving outcome prediction in various clinical scenarios [10, 11]. In particular, the MIMIC-III and MIMIC-IV databases have been extensively used to develop machine learning models for mortality prediction in critically ill patients. For sepsis populations, Hou et al. applied an XGBoost model to MIMIC-III sepsis-3 patients and demonstrated 30 day mortality prediction compared with conventional scoring systems [12]. In parallel, machine learning models have also been developed in ICU patients with diabetes, including type 2 diabetes mellitus, to predict mortality and readmission using high-dimensional electronic health record data [13]. These works collectively highlight both the feasibility and the value of applying machine learning to large critical care datasets such as MIMIC.

Despite these advances, only a limited number of studies have specifically examined ICU patients with both sepsis and diabetes. Existing work in this subgroup has largely focused on individual biomarkers or composite indices, and has primarily used Cox or logistic regression models to examine associations with short term and long term mortality [14]. These studies provide important evidence regarding particular prognostic markers, but they do not comprehensively evaluate and compare multiple machine learning algorithms using a broad panel of routinely available clinical variables, nor do they systematically benchmark their performance against established severity scores like SOFA.

To address these gaps, we conducted a retrospective cohort study using the MIMIC-IV database to develop and validate machine learning models for predicting in-hospital mortality in ICU patients with both sepsis and diabetes mellitus. We systematically compared six commonly used algorithms: logistic regression (LR), K-nearest neighbors (KNN), random forest (RF), gradient boosting decision tree (GBDT), XGBoost and LightGBM with the SOFA score in terms of discrimination, calibration, and precision recall performance. Furthermore, to enhance clinical interpretability and facilitate potential bedside use, we applied SHAP (SHapley Additive exPlanations) analysis to the best performing model to elucidate the relative importance and directional impact of key predictors in this specific patient population.

## Methods

### Data source

We conducted a retrospective analysis of electronic medical records from 7716 patients, utilizing data from the Medical Information Mart for Intensive Care (MIMIC-IV) database [15]. This extensive clinical resource contains medical records for over 65,000 patients who received treatment in the emergency department or intensive care unit at the Beth Israel Deaconess Medical Center in Boston, Massachusetts, between 2008 and 2022. Since this research involved secondary analysis of a publicly accessible third-party database, institutional review board approval was not needed.

### Inclusion and exclusion criteria

We included adult patients admitted to the ICU with a diagnosis of sepsis according to Sepsis-3 criteria. Sepsis was defined as a suspected or documented infection with an acute increase in SOFA score ≥ 2 points. Pre-existing diabetes mellitus was identified using ICD-9-CM or ICD-10-CM codes, with diagnosis confirmed prior to ICU admission. Only the first ICU admission during the study period was considered for each patient. Exclusion criteria encompassed patients below 18 years and pregnant women, because these populations have distinct pathophysiology, treatment pathways, and outcome profiles compared with the general adult ICU population. Patients diagnosed with AIDS, malignant cancer, or metastatic solid tumors were excluded because these conditions are associated with markedly different baseline mortality risks and competing prognostic trajectories, which could confound the relationship between sepsis, diabetes, and in-hospital mortality. We also excluded patients with an ICU length of stay < 24 h to ensure a sufficient observation window for collecting early clinical data and to avoid including short stay cases related to postoperative monitoring or early palliative decisions. Finally, patients with more than 20% missing data in the variables of interest were excluded to preserve data quality and to limit the extent of imputation required in the analysis.

### Data extraction

We extracted comprehensive clinical data from the MIMIC-IV database, focusing on the first 24 h following ICU admission for each patient. The first 24-hour window was chosen for three reasons. First, it reflects a clinically meaningful early assessment period, during which intensivists typically make key decisions regarding triage, organ support, and resource allocation. Second, restricting predictors to this interval allows our models to be used for early risk stratification before substantial post admission deterioration, therapeutic interventions, and complications occur, thereby reducing the risk of incorporating intermediate outcomes along the causal pathway. Third, a 24-hour window provides a practical balance between information content and data completeness: shorter windows (e.g., 6–12 h) would miss many routinely ordered laboratory tests, whereas longer windows (e.g., 48 h) would capture later trajectories at the cost of delayed prediction and potential label leakage.

The extracted information encompassed demographics (age, sex, body mass index, and ethnicity), comorbidities (including congestive heart failure, hypertension, liver diseases), laboratory test results (such as creatinine, electrolytes, and lactate), and disease severity indicator (SOFA score). For laboratory parameters with multiple measurements, we included both maximum and minimum values in our analysis, while for SOFA scores, we used the first recorded value. The dataset was randomly split into training (70%) and validation (30%) cohorts. The dataset was preprocessed by handling missing values, scaling features, and encoding categorical variables where appropriate. To address missing data, we employed multiple imputation techniques based on the random forest algorithm with 50 estimators, a method that has demonstrated efficacy in handling missing values in clinical datasets [16].

### Statistical analysis

Descriptive statistics were employed to summarize patient characteristics. We expressed continuous variables as median values accompanied by their interquartile ranges (IQR) and conducted comparisons using the Mann-Whitney U test. For categorical variables, we presented them as frequencies and percentages, with comparisons performed via chi-square tests or Fisher’s exact tests when appropriate.We conducted all statistical analyses and machine learning model implementations using Python version 3.10. The threshold for statistical significance was set at a two-sided p-value of less than 0.05 for all analyses.

We generated Receiver Operating Characteristic (ROC) curves and Precision–Recall (PR) curves and calculated the area under each curve (AUC-ROC and AUC-PR) to evaluate the models’ overall discriminative ability. To further characterize performance at clinically relevant operating points, we converted predicted probabilities into binary classifications using model-specific probability thresholds determined in the training cohort. For each model, the optimal cut-off was chosen as the threshold that maximized Youden’s index (sensitivity + specificity − 1) in the training data. These fixed thresholds were then applied to the validation cohort to construct confusion matrices and to calculate accuracy, sensitivity (recall), specificity, precision, and F1-score for each model. We calculated 95% confidence intervals for AUC-ROC and AUC-PR using bootstrapping with 1000 iterations to assess the stability of our results. To provide insights into the relative importance of predictor variables, we applied SHAP values to the best-performing model [17, 18].

### Implementation of machine learning models

We implemented six machine learning algorithms to develop predictive models for in-hospital mortality. Additionally, we compared these models with the traditional SOFA score. To mitigate overfitting, we employed a rigorous hyperparameter tuning process. The training cohort was divided into five partitions using k-fold cross validation, with four-fifths used for training and the remainder for validation. Grid search with five-fold cross-validation was utilized to identify the most appropriate hyperparameters for each model. Two key requirements for fine-tuned hyperparameters were: (1) the training loss was minimized across different combinations of model hyperparameters, and (2) the log loss in the validation part was less than -log 0.5 and slightly greater than that in the training part.

## Results

### Baseline characteristics

From the MIMIC-IV database, we initially identified 31,911 patients diagnosed with sepsis. Among these, 9982 patients had a concurrent diagnosis of diabetes mellitus. We applied our exclusion criteria sequentially: 992 patients were excluded due to an ICU stay less than 24 h, leaving 8,990 patients. Further exclusions were made for patients with AIDS (39 patients) and those diagnosed with malignant cancer or metastatic solid tumor (1,001 patients). Finally, 234 patients were excluded due to missing data exceeding 20% of the variables of interest. After applying all exclusion criteria, our final study cohort consisted of 7,716 patients with both sepsis and diabetes mellitus. The patient selection process is illustrated in Fig. 1.

**Fig. 1:**
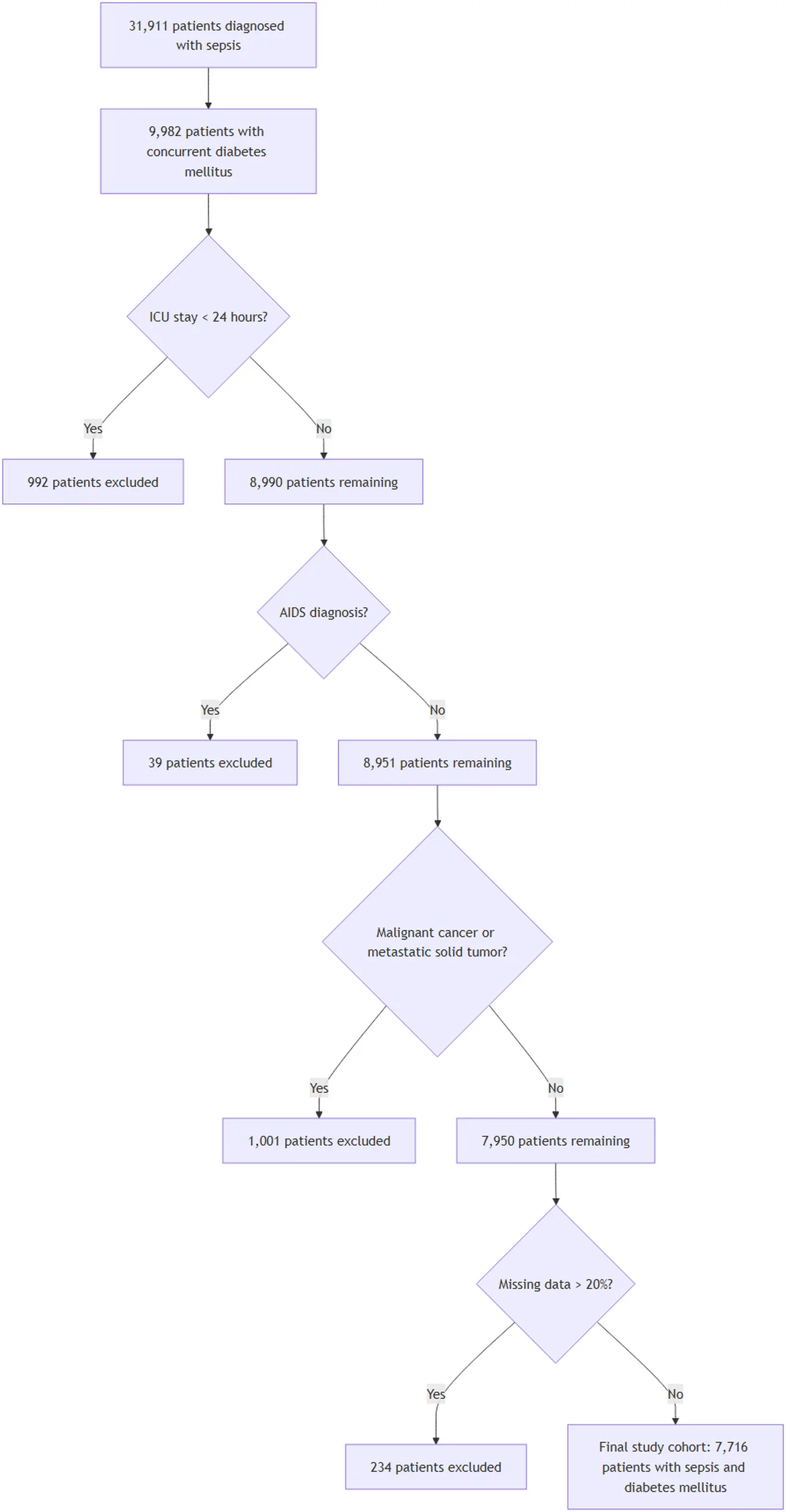
Flowchart of patient selection process for diabetic sepsis cohort from MIMIC-IV database

Within this cohort, 1357 (17.6%) patients died during their hospital stay. The cohort was randomly divided into a training set (*n* = 5401, 70%) and a validation set (*n* = 2315, 30%). Detailed baseline characteristics of the patients in both the training and validation cohorts are presented in Table 1. The distribution of demographics, comorbidities, laboratory values, and clinical parameters were similar between the two cohorts, indicating a successful randomization process. The proportion of patients who experienced in-hospital mortality was comparable between the training (17.6%) and validation (17.6%) cohorts. The overall proportion of missing data for the included predictors was low to moderate, and the distribution of missing values across variables is presented in Supplementary Table S1. Most demographic and comorbidity variables had minimal missingness, whereas a subset of laboratory tests (e.g., lactate and albumin) exhibited higher but still acceptable rates of missing data prior to imputation.

**Table 1:** Baseline characteristics of included patients

Characteristics	Total (*n* = 7716)	Training cohort (*n* = 5401)	Validation cohort (*n* = 2315)	*P* value
**Demographic variables**				
Age (years)	70 (61,78)	70 (61,78)	69 (60,78)	0.464
Gender, n (%)				0.242
Male Female	4290 (55.6%) 3426 (44.4%)	2979 (55.2%) 2422 (44.8%)	1311 (56.6%) 1004 (43.4%)	
BMI (kg/m2)	31.3 (28.0,34.2)	31.4 (28.1,34.2)	31.2 (27.7,34.2)	0.155
Marital status, n (%)				0.639
Single Married Other	1909 (24.7%) 3471 (45.0%) 2336 (30.3%)	1342 (24.8%) 2411 (44.6%) 1648 (30.5%)	567 (24.5%) 1060 (45.8%) 688 (29.7%)	
Ethnicity, n (%)				0.936
White Other	4486 (58.1%) 3230 (41.9%)	3138 (58.1%) 2263 (41.9%)	1348 (58.2%) 967 (41.8%)	
Admission Type, n (%)				0.012
Emergency Elective Urgent	5406 (72.4%) 285 (3.8%) 1772 (23.7%)	3826 (73.3%) 204 (3.9%) 1188 (22.8%)	1580 (70.4%) 81 (3.6%) 584 (26.0%)	
**Comorbidities**				
Myocardial infarction Congestive heart failure Peripheral vascular disease Cerebrovascular disease Dementia Chronic pulmonary disease Rheumatic disease Peptic ulcer disease Mild liver disease Paraplegia Renal disease	2075 (26.9%) 3371 (43.7%) 1115 (14.5%) 1353 (17.5%) 455 (5.9%) 2216 (28.7%) 240 (3.1%) 245 (3.2%) 1022 (13.2%) 439 (5.7%) 2981 (38.6%)	1409 (26.1%) 2352 (43.5%) 790 (14.6%) 918 (17.0%) 307 (5.7%) 1547 (28.6%) 153 (2.8%) 166 (3.1%) 697 (12.9%) 308 (5.7%) 2078 (38.5%)	666 (28.8%) 1019 (44.0%) 325 (14.0%) 435 (18.8%) 148 (6.4%) 669 (28.9%) 87 (3.8%) 79 (3.4%) 325 (14.0%) 131 (5.7%) 903 (39.0%)	0.016 0.722 0.524 0.062 0.247 0.841 0.038 0.479 0.191 0.982 0.679
**Laboratory tests**				
Aniongap_min Aniongap_max Albumin_min Albumin_max Bun_min Bun_max Creatinine_min Creatinine_max Chloride_min Chloride_max Glucose_min Glucose_max Lactate_min Lactate_max Platelets_min Platelets_max Hematocrit_min Hematocrit_max Hemoglobin_min Hemoglobin_max Potassium_min Potassium_max PTT_min PTT_max INR_min INR_max PT_min PT_max Sodium_min Sodium_max WBC_min WBC_max Calcium_min Calcium_max ALT_min ALT_max ALP_min ALP_max AST_min AST_max **CCI** **SOFA**	13.0 (11.0,15.0) 16.0 (14.0,20.0) 3.1 (2.8,3.4) 3.2 (2.9,3.5) 24.0 (15.0,41.0) 30.0 (19.0,50.0) 1.2 (0.8,1.9) 1.4 (1.0,2.4) 102.0 (97.0,106.0) 106.0 (101.0,110.0) 127.5 (102.0,162.0) 187.0 (138.0,265.0) 1.5 (1.1,2.0) 2.4 (1.6,3.6) 171.0 (120.0,237.0) 212.0 (156.0,286.0) 28.9 (24.9,33.4) 33.6 (29.8,38.0) 9.4 (8.1,10.9) 10.9 (9.5,12.4) 4.0 (3.6,4.4) 4.6 (4.2,5.2) 29.4 (26.1,33.8) 34.1 (28.9,46.9) 1.2 (1.1,1.4) 1.4 (1.2,1.7) 13.8 (12.3,15.7) 15.2 (13.3,18.3) 137.0 (134.0,139.0) 140.0 (137.0,142.0) 10.1 (7.3,13.5) 14.1 (10.2,19.0) 8.1 (7.7,8.6) 8.5 (8.2,9.0) 57.0 (21.0,93.0) 62.0 (22.0,119.3) 104.5 (77.0,121.0) 116.0 (84.0,130.0) 91.3 (29.0,145.7) 110.0 (33.0,223.6) 6.0 (5.0,8.0) 3.0 (2.0,5.0)	13.0 (11.0,15.0) 16.0 (14.0,20.0) 3.1 (2.8,3.4) 3.2 (2.9,3.5) 24.0 (15.0,41.0) 30.0 (19.0,49.0) 1.2 (0.8,1.9) 1.4 (1.0,2.4) 102.0 (97.0,106.0) 106.0 (101.0,110.0) 128.0 (102.0,162.0) 188.0 (139.0,266.0) 1.5 (1.1,2.0) 2.4 (1.6,3.6) 171.0 (121.0,237.0) 213.0 (156.0,286.0) 28.8 (24.8,33.3) 33.5 (29.8,38.0) 9.4 (8.1,10.9) 10.9 (9.5,12.4) 4.0 (3.6,4.4) 4.6 (4.2,5.2) 29.4 (26.1,33.8) 34.2 (28.9,47.0) 1.2 (1.1,1.4) 1.4 (1.2,1.7) 13.7 (12.3,15.7) 15.2 (13.2,18.2) 137.0 (134.0,139.0) 140.0 (137.0,142.0) 10.1 (7.3,13.5) 14.1 (10.3,19.1) 8.1 (7.7,8.6) 8.6 (8.2,9.0) 58.0 (21.0,93.1) 62.4 (23.0,120.4) 105.0 (77.0,121.1) 116.2 (85.0,130.0) 96.0 (29.0,146.1) 113.0 (33.0,225.0) 6.0 (5.0,8.0) 3.0 (2.0,5.0)	13.0 (11.0,15.0) 16.0 (13.0,20.0) 3.1 (2.8,3.4) 3.2 (2.9,3.5) 24.0 (16.0,42.0) 31.0 (19.0,51.0) 1.2 (0.8,2.0) 1.4 (1.0,2.5) 102.0 (97.0,106.0) 106.0 (101.0,110.0) 127.0 (100.0,161.5) 185.0 (136.0,260.5) 1.5 (1.1,1.9) 2.4 (1.6,3.5) 170.0 (119.0,237.0) 210.0 (155.0,288.0) 29.0 (25.1,33.6) 33.6 (29.8,38.0) 9.5 (8.1,11.0) 11.0 (9.6,12.4) 4.0 (3.6,4.4) 4.6 (4.2,5.1) 29.4 (26.2,33.9) 33.9 (28.9,46.6) 1.3 (1.1,1.4) 1.4 (1.2,1.7) 13.9 (12.4,15.8) 15.3 (13.3,18.4) 137.0 (134.0,139.0) 140.0 (137.0,142.0) 10.0 (7.2,13.5) 13.8 (9.9,18.8) 8.1 (7.7,8.6) 8.5 (8.1,9.0) 55.0 (20.0,92.6) 61.0 (22.0,116.3) 104.0 (75.0,121.0) 115.0 (82.0,130.0) 83.0 (29.0,144.7) 105.0 (33.0,219.7) 6.0 (5.0,8.0) 3.0 (2.0,5.0)	0.805 0.83 0.554 0.937 0.221 0.227 0.288 0.496 0.694 0.632 0.321 0.081 0.015 0.11 0.861 0.409 0.189 0.765 0.121 0.779 0.497 0.48 0.695 0.754 0.154 0.508 0.135 0.51 0.722 0.769 0.407 0.079 0.348 0.066 0.191 0.195 0.214 0.296 0.48 0.204 0.255 0.166

Abbreviations: BMI: Body Mass Index; CCI: Charlson Comorbidity Index; SOFA: Sequential Organ Failure Assessment; BUN: Blood Urea Nitrogen; PTT: Partial Thromboplastin Time; INR: International Normalized Ratio; PT: Prothrombin Time; WBC: White Blood Cell; ALT: Alanine Aminotransferase; ALP: Alkaline Phosphatase; AST: Aspartate Aminotransferase

### Evaluation of model performance

Six machine learning models were developed to predict in-hospital mortality in patients with sepsis and diabetes mellitus: Logistic Regression (LR), K-Nearest Neighbors (KNN), Random Forest (RF), Gradient Boosting Decision Tree (GBDT), XGBoost, and LightGBM. These models were compared with the traditional SOFA score. Figure 2 presents the ROC curves for all models. The Gradient Boosting Decision Tree model demonstrated the best performance with an area under the ROC curve of 0.792 (95% CI: 0.769–0.814), followed closely by XGBoost (AUC = 0.785, 95% CI: 0.762–0.807) and LightGBM (AUC = 0.784, 95% CI: 0.762–0.806). All machine learning models outperformed the SOFA score (AUC = 0.617, 95% CI: 0.587–0.647). The performance of other models was as follows: Random Forest (AUC = 0.764, 95% CI: 0.740–0.788), Logistic Regression (AUC = 0.705, 95% CI: 0.680–0.731), and K-Nearest Neighbors (AUC = 0.669, 95% CI: 0.640–0.695). The non-overlapping confidence intervals between the top-performing models and the SOFA score indicate statistically significant improvements in predictive ability. Figure 3 illustrates the Precision-Recall curves for all models. The GBDT model again showed superior performance with the highest area under the Precision-Recall curve (AP = 0.475), followed by XGBoost (AP = 0.458) and LightGBM (AP = 0.461). These results further confirm the superior predictive capability of the tree-based ensemble models in this context. The calibration curve plot (Fig. 4) indicated good agreement between the predicted probabilities of in-hospital mortality derived from machine learning models and the actual outcomes. The GBDT, XGBoost, and LightGBM models showed particularly good calibration, closely following the ideal calibration line. Figure 5 presents the confusion matrices for all six machine learning models, providing a detailed visualization of their classification performance in terms of true positives, true negatives, false positives, and false negatives. In addition, threshold-dependent metrics at the Youden-optimal cut-offs derived from the training cohort are presented in Table 2.

**Fig. 2:**
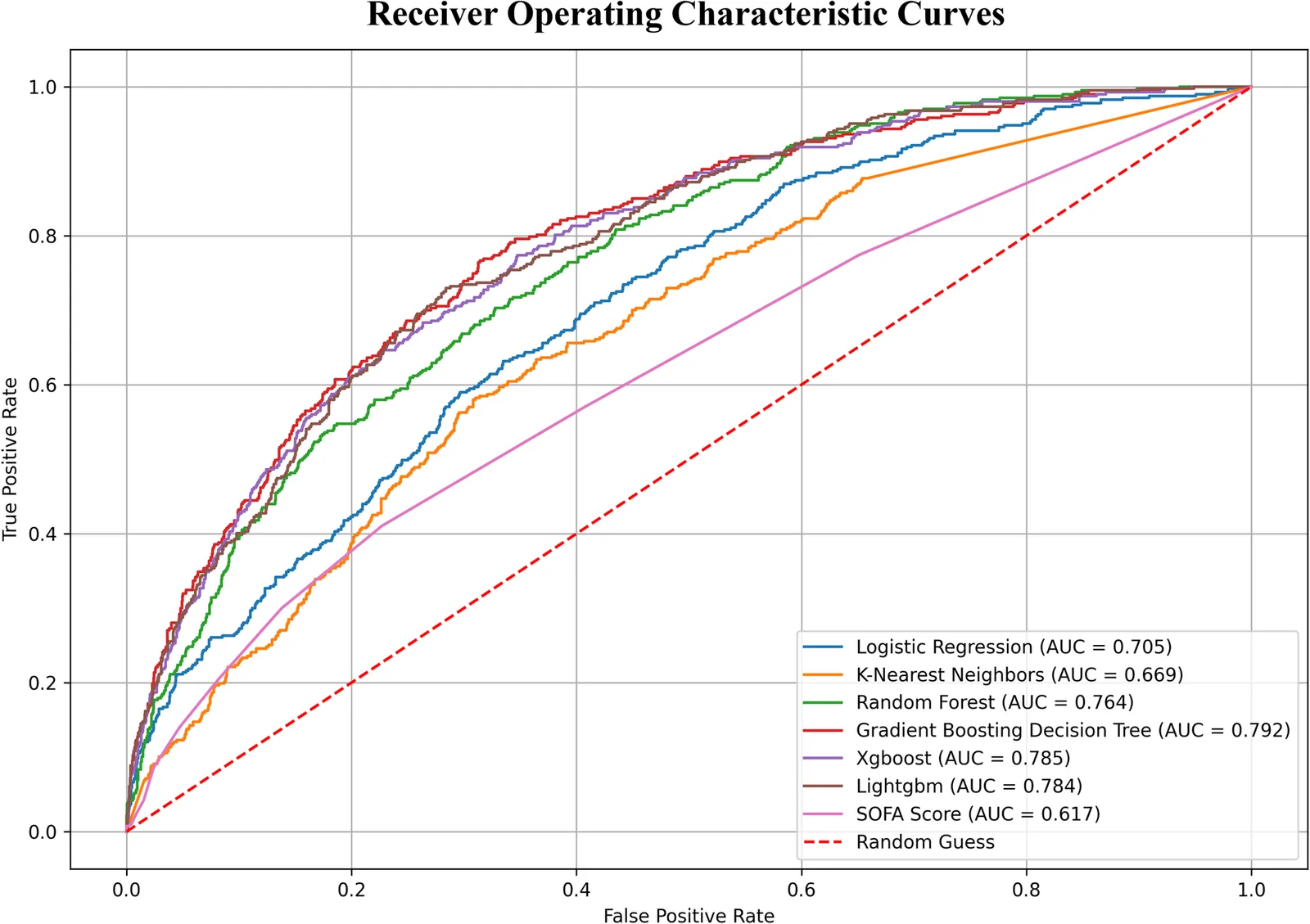
ROC curves comparing machine learning models and SOFA score for predicting in-hospital mortality

**Fig. 3:**
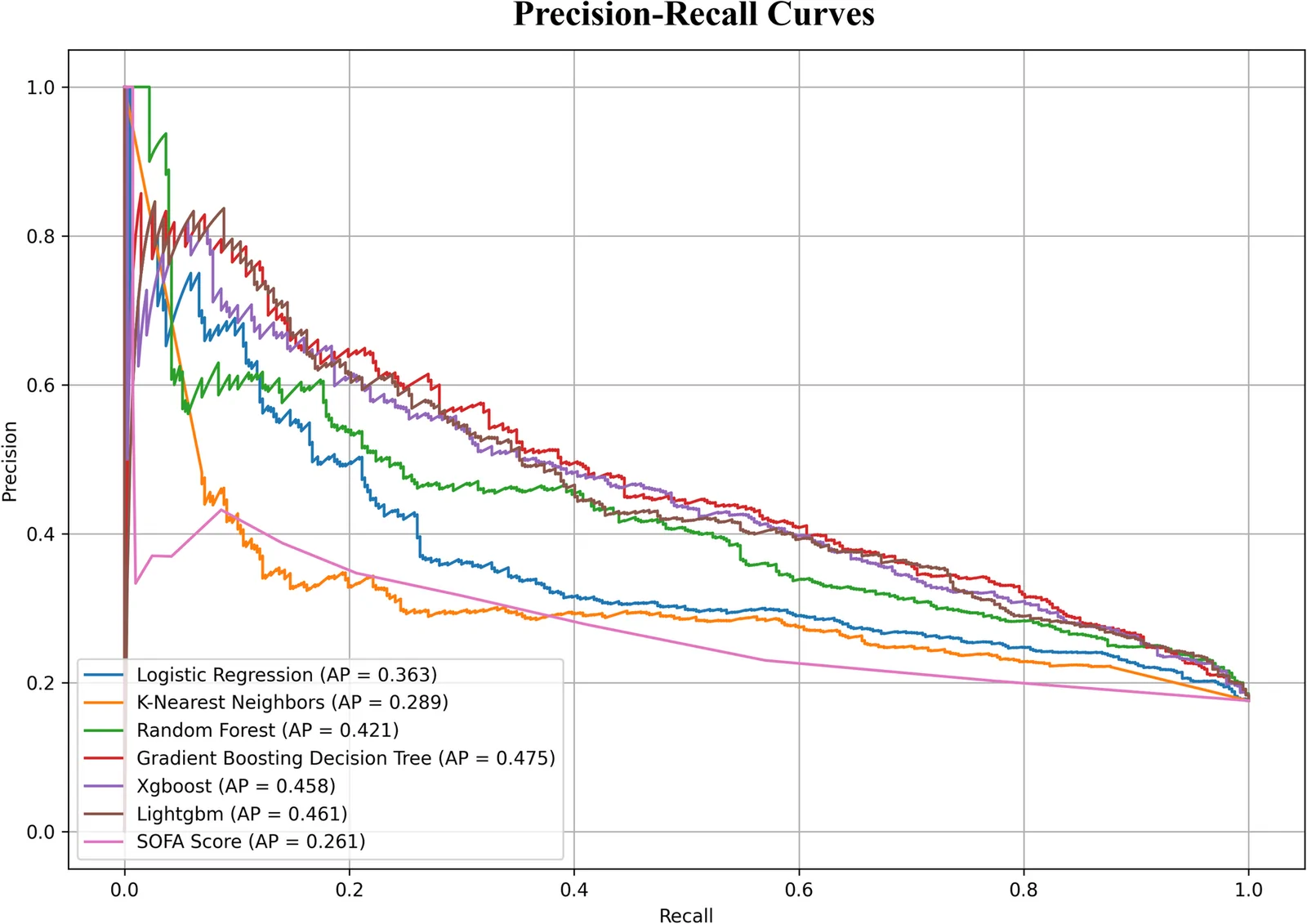
Precision-recall curves of machine learning models for mortality prediction

**Fig. 4:**
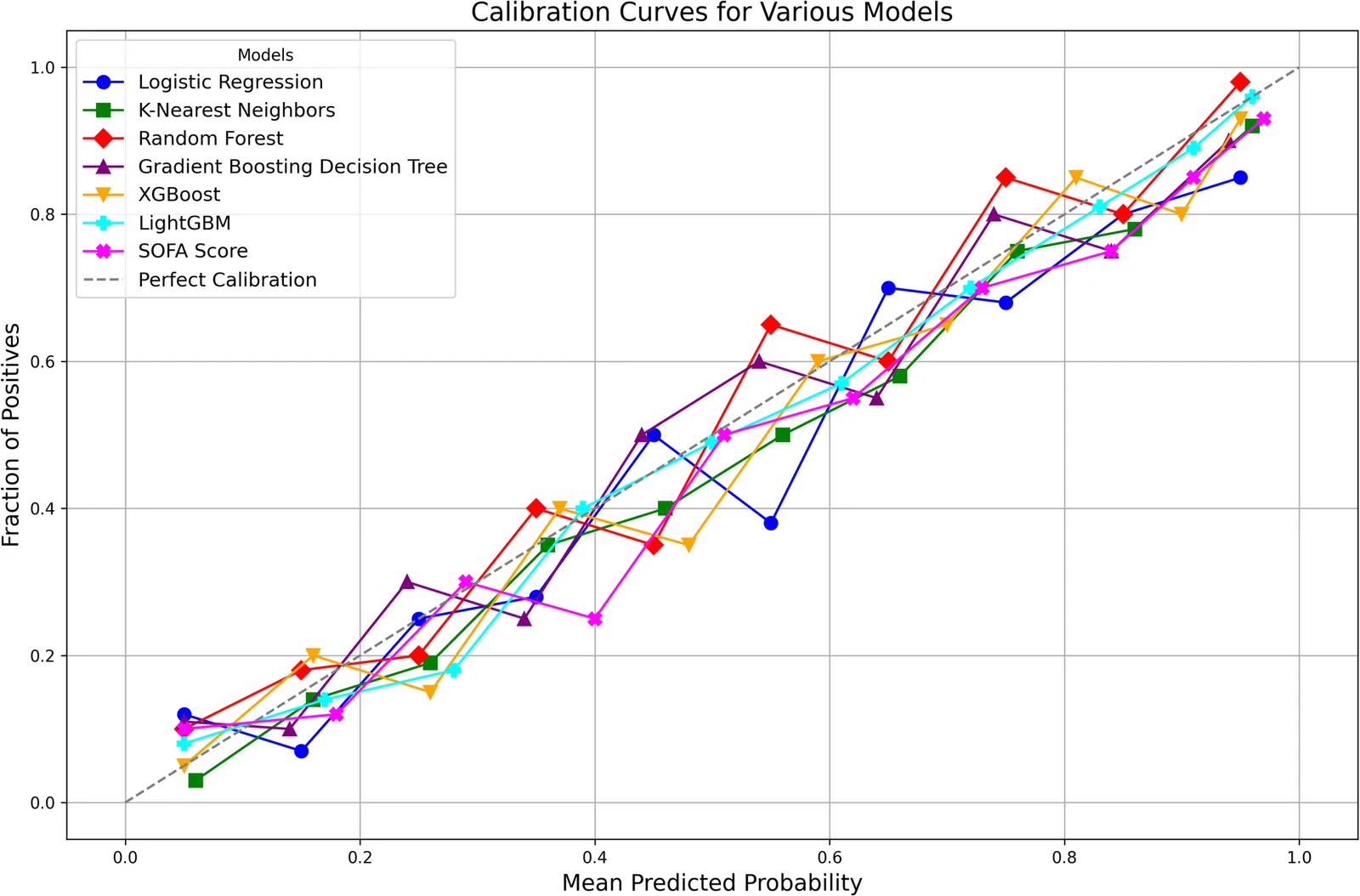
Calibration curves of machine learning models for in-hospital mortality prediction

**Fig. 5:**
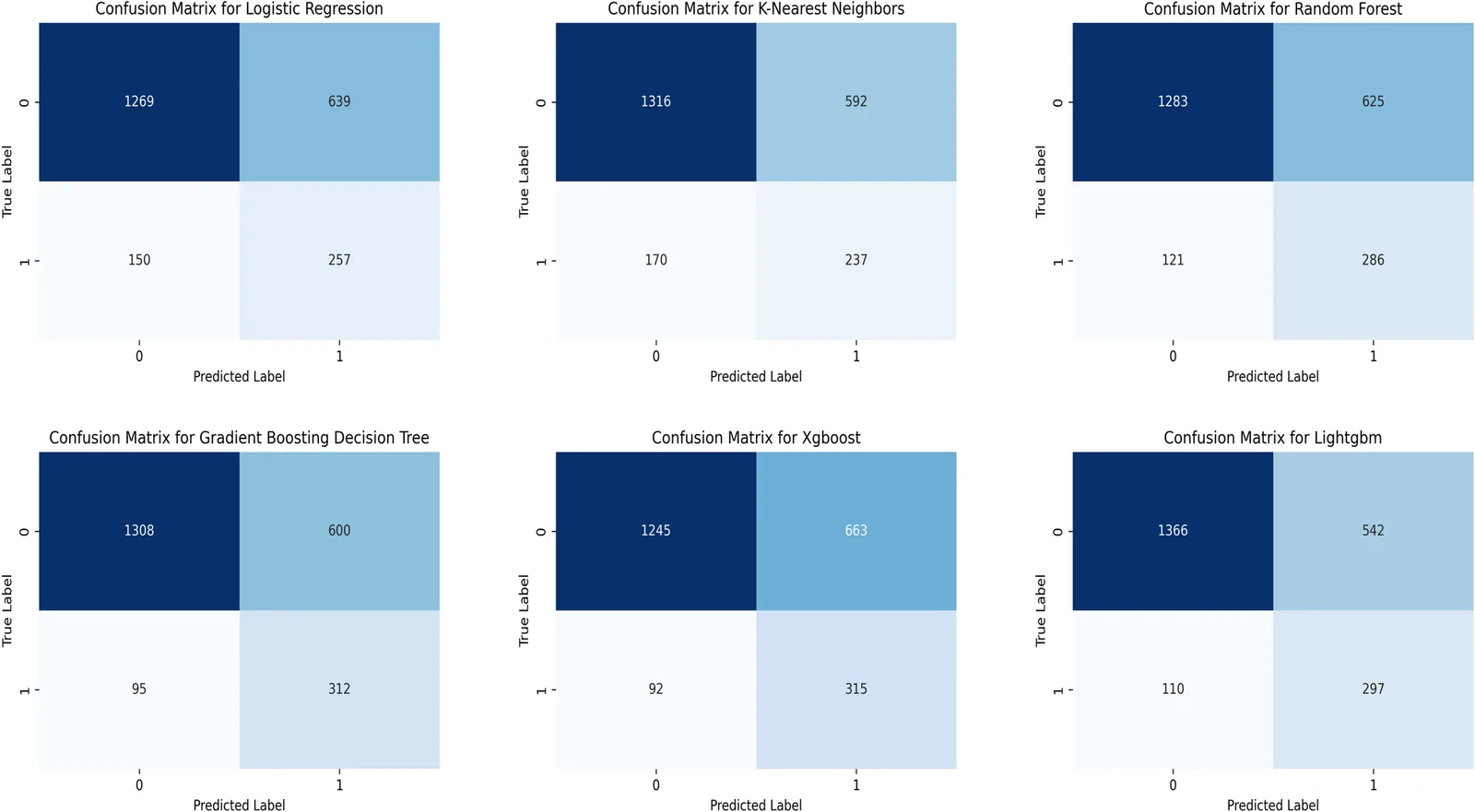
Confusion matrices for six machine learning models predicting in-hospital mortality

**Table 2:** Threshold dependent performance metrics of machine learning models in the validation cohort

Model	Accuracy	Sensitivity	Specificity	Precision	F1-score
Logistic Regression	0.659	0.631	0.665	0.287	0.394
K-Nearest Neighbors	0.671	0.582	0.690	0.286	0.383
Random Forest	0.678	0.703	0.672	0.314	0.434
Gradient Boosting Decision Tree	0.700	0.767	0.686	0.342	0.473
XGBoost	0.674	0.774	0.653	0.322	0.455
LightGBM	0.718	0.730	0.716	0.354	0.477

### Model interpretation

Figure 6 presents the SHAP values for the GBDT model, illustrating the most influential predictors for in-hospital mortality among ICU patients with sepsis and diabetes mellitus. The Charlson Comorbidity Index (CCI) was identified as the top predictor, with higher scores indicating increased mortality risk. Age also emerged as a key factor, with older patients showing a higher likelihood of mortality. Elevated SOFA score, maximum lactate levels, higher, increased maximum creatinine levels, and prolonged maximum prothrombin time were also important predictors, all associated with increased risk of in-hospital mortality. The SHAP analysis also highlighted lower minimum albumin levels and decreased minimum platelet counts as significant factors linked to higher mortality. The relationship between maximum white blood cell (WBC) count and mortality was complex, indicating that both very high and very low counts could be associated with increased mortality risk. Additionally, minimum hemoglobin levels showed a nuanced influence on mortality risk. These SHAP values provide insight into the hierarchical importance of clinical features contributing to the GBDT model’s predictions.

**Fig. 6:**
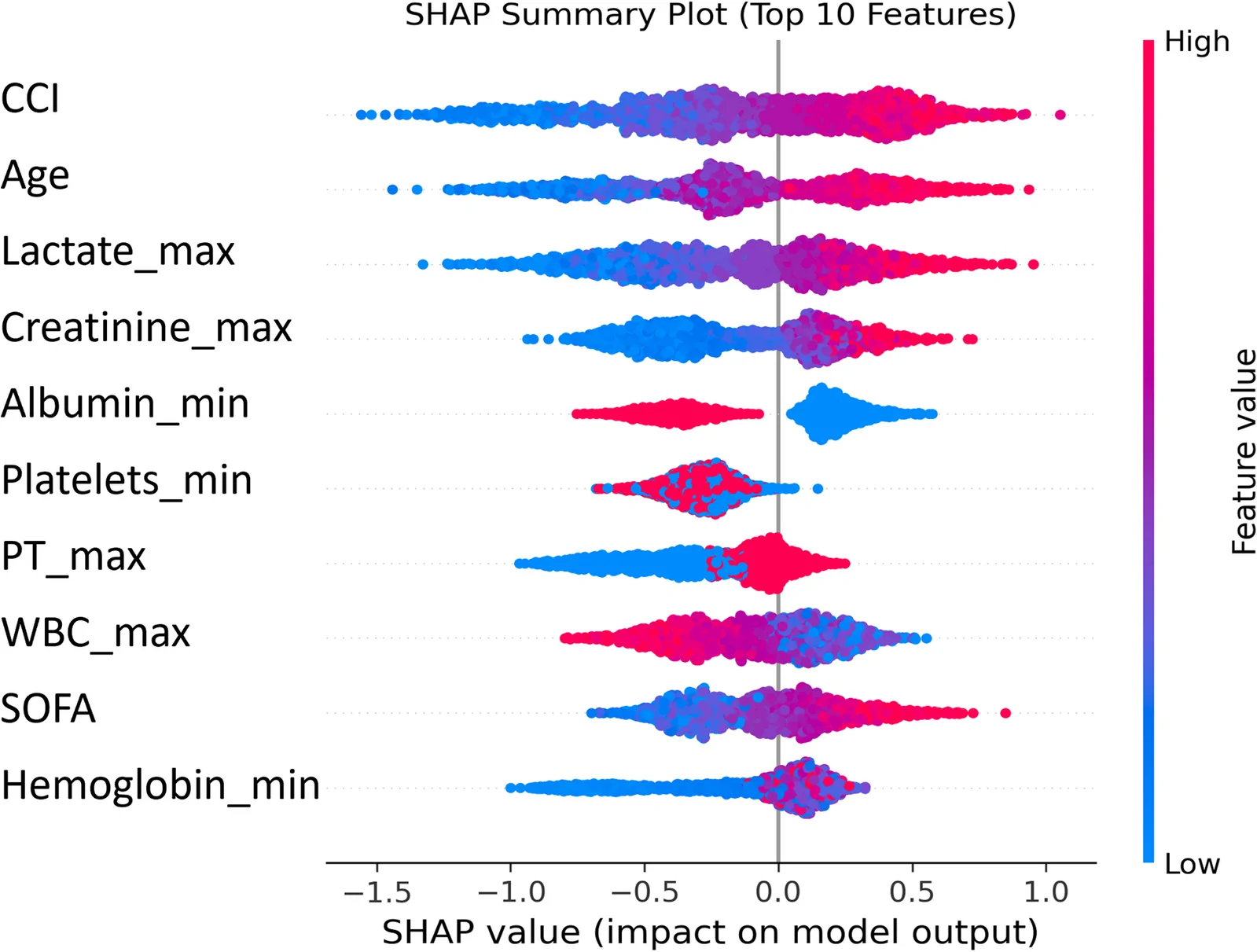
SHAP analysis of top predictors for in-hospital mortality in GBDT model

## Discussion

This study demonstrates the superior performance of machine learning models, particularly the GBDT model, in predicting in-hospital mortality for sepsis patients with diabetes mellitus. Our findings underscore the potential of advanced analytical techniques to enhance prognostication in this complex patient population, potentially leading to improved clinical decision-making and resource allocation. The GBDT model achieved the highest predictive performance (AUC 0.792), significantly outperforming the traditional SOFA score (AUC 0.617). This substantial improvement in predictive accuracy aligns with previous studies that have shown the potential of machine learning in sepsis prognostication [10, 11]. However, our study is unique in its focus on the specific subpopulation of sepsis patients with diabetes, addressing an important gap in the literature. In contrast to previous researches, our study focuses on a well defined subgroup of ICU patients with both sepsis and diabetes, uses a comprehensive set of routinely available early ICU variables, systematically benchmarks six machine learning algorithms against the SOFA score, and employs SHAP analysis to provide a transparent ranking and interpretation of key predictors in this clinically important population. Our SHAP analysis revealed critical insights into the most influential predictors of in-hospital mortality in our model. The top 10 predictors identified were: CCI, age, maximum lactate, maximum creatinine, minimum albumin, minimum platelet count, maximum prothrombin time, maximum WBC, SOFA score, and minimum hemoglobin. The CCI and age emerged as the strongest predictors, underscoring the impact of comorbidity burden and advanced age on outcomes in diabetic sepsis [19–21]. Elevated lactate and creatinine reflected the importance of tissue hypoperfusion and renal dysfunction as key determinants of mortality [22–24]. Lower albumin levels highlighted the prognostic relevance of nutritional status [25], thrombocytopenia underscored the role of hematologic dysfunction [26], and prolonged prothrombin time reflected the impact of coagulation abnormalities on sepsis outcomes [27]. Finally, extremes of white blood cell count and reduced hemoglobin were also associated with higher mortality risk, indicating the complex interplay between inflammatory responses, anemia, and prognosis in critically ill patients [28, 29].

The superior performance of our machine learning models compared with the SOFA score suggests that these approaches capture more nuanced relationships in the data, which is particularly relevant in diabetic sepsis where complex physiological interactions may not be fully reflected by traditional scores [1]. The SHAP-based explanations reflect statistical patterns in the model that may at times appear counterintuitive clinically, highlighting the non-linear and multifactorial nature of the associations between clinical parameters and mortality risk in this population. Taken together, these findings indicate that integrating such models into clinical workflows has the potential to enhance risk stratification and support more timely, targeted interventions. However, realizing these potential benefits in practice requires careful consideration of how the models perform at clinically relevant decision thresholds. In this high-risk context, false negatives, where truly high risk patients are classified as low risk, may delay recognition of deterioration and escalation of organ support. False positives, where lower-risk patients are flagged as high risk, can lead to unnecessary alarms, intensified monitoring, and possible overtreatment, with implications for resource use and alarm fatigue. In this study, we therefore reported confusion matrices and threshold dependent metrics based on model-specific cut-offs that maximized Youden’s index, providing a statistically balanced trade-off between sensitivity and specificity. This operating point is not intended as a definitive clinical threshold; in practice, threshold selection should be guided by local resource constraints and risk tolerance, and in many intensive care units a more sensitive operating point that accepts more false positives in order to reduce missed high risk patients may be preferable. Prospective implementation studies and decision-curve analyses will be needed to identify clinically acceptable thresholds for real world use.

From a practical perspective, the variables used in our models are routinely collected in the ICU which facilitates integration into existing electronic health record systems. In a real world setting, the model could function as an automated decision-support tool that generates an individualized in-hospital mortality risk estimate once 24-hour ICU data are available and displays this information during daily rounds to support early risk stratification and targeted intensification of monitoring and organ support. Our model is intended as an adjunct to, rather than a replacement for, clinical judgment, and any implementation should be accompanied by prospective evaluation of its impact on workflow and patient outcomes. At present, these results should be regarded as proof of concept, and further external validation and prospective impact studies are required before routine deployment in ICU decision-support systems.

Despite this practical potential, several important challenges and sources of bias must be acknowledged. A key challenge is the interpretability of complex models such as GBDT [30]. Although the use of SHAP values provides insight into feature contributions, further work is needed to make these models sufficiently transparent and acceptable to clinicians. Generalizability is another major limitation. All analyses were conducted using the single-center MIMIC-IV database, and case-mix, practice patterns, and data collection processes may differ substantially in other institutions and healthcare systems. Our models were evaluated only by internal validation with a random split into training and validation cohorts, and their performance and calibration may change when applied to external populations. Accordingly, the current results should be regarded as hypothesis-generating and primarily applicable to settings similar to the MIMIC-IV cohort rather than as evidence of broad generalizability. In line with current recommendations for prediction model research [31], multi-center external validation is essential before any clinical implementation. We are currently prospectively collecting data on ICU patients with sepsis and diabetes from our institution and collaborating centers to construct an independent validation cohort. A further limitation arises from the retrospective observational design and reliance on routinely collected ICU data. Pre-ICU diabetes control, infection source, microbiological characteristics, and detailed treatment strategies, including antibiotic timing, fluid resuscitation, vasopressor management, and glucose control protocols, may influence both predictor values and mortality risk but were not fully incorporated into our models. In addition, differences in clinical management across ICU units and over time within the MIMIC-IV cohort may introduce selection and information bias. As a consequence, our models should be interpreted as capturing statistical associations rather than causal relationships, and caution is required to avoid inferring causal effects of individual predictors on mortality from these results.

Despite these limitations, our study provides evidence that machine learning can improve prognostication in sepsis patients with diabetes. The clear improvement in predictive performance compared with the SOFA score suggests that these approaches can support more refined risk stratification and may contribute to better clinical decision-making in this complex population. Future research should prioritize multi-center external validation, formal assessment of the impact of these models on clinical processes and outcomes when embedded in real-world workflows, and further work to enhance interpretability for end users, including prospective studies that evaluate real-time use at the bedside. In conclusion, our study shows that machine learning models, particularly the Gradient Boosting Decision Tree model, outperform the SOFA score in predicting in-hospital mortality among sepsis patients with diabetes. The SHAP analysis provided important insights into the interplay of comorbidities, organ dysfunction, and laboratory abnormalities that drive mortality risk in this group, highlighting both established and less expected predictors. With continued refinement, rigorous external validation, and prospective evaluation, such models may become valuable tools in the management of sepsis in patients with diabetes and help improve outcomes in critical care settings.

## Conclusion

In this single-center cohort of ICU patients with sepsis and diabetes, machine learning models—particularly the Gradient Boosting Decision Tree model—outperformed the SOFA score in predicting in-hospital mortality. SHAP analysis provided clinically plausible insights into key predictors, suggesting that these approaches may support more refined risk stratification in this high-risk population. However, the retrospective design and single-center MIMIC-IV data limit generalizability, and the models should currently be regarded as hypothesis-generating. Future work should include multi-center external validation and prospective evaluation of real-time use at the bedside to determine the practical utility, safety, and impact of these models in routine critical care practice.

## Electronic Supplementary Material

Below is the link to the electronic supplementary material.

Supplementary Material 1

## Data Availability

The research involved secondary analysis of the publicly accessible and de-identified MIMIC-IV database. Access to the database was granted after completion of the Protecting Human Research Participants training.

## Abbreviations

AIDS: Acquired Immune Deficiency Syndrome
ALP: Alkaline Phosphatase
ALT: Alanine Aminotransferase
AP: Average Precision
AST: Aspartate Aminotransferase
AUC: Area Under the Curve
BMI: Body Mass Index
BUN: Blood Urea Nitrogen
CCI: Charlson Comorbidity Index
CI: Confidence Interval
GBDT: Gradient Boosting Decision Tree
ICD-10-CM: International Classification of Diseases, 10th Revision, Clinical Modification
ICD-9-CM: International Classification of Diseases, 9th Revision, Clinical Modification
ICU: Intensive Care Unit
INR: International Normalized Ratio
IQR: Interquartile Range
KNN: K-Nearest Neighbors
LR: Logistic Regression
LightGBM: Light Gradient Boosting Machine
MIMIC-IV: Medical Information Mart for Intensive Care IV
PR: Precision–Recall
PT: Prothrombin Time
PTT: Partial Thromboplastin Time
RF: Random Forest
ROC: Receiver Operating Characteristic
SHAP: SHapley Additive exPlanations
SOFA: Sequential Organ Failure Assessment
WBC: White Blood Cell
XGBoost: Extreme Gradient Boosting
