# Supplementary material for: Application of machine learning prediction models for in-hospital mortality in diabetic sepsis patients: a study based on the MIMIC IV database
Source: BMC Med Inform Decis Mak. 2026 Oct 22;26:426. doi: 10.1186/s12911-026-03580-4 (PMC13688817; doi:10.1186/s12911-026-03580-4)
Supplement: Supplementary file 1 [file 12911_2026_3580_MOESM1_ESM.docx]

| **Category** | **Variable** | **Missing values, n** | **Missing values, %** |
| --- | --- | --- | --- |
| Demographic variables | Age | 0 | 0.0 |
| Demographic variables | Gender | 0 | 0.0 |
| Demographic variables | BMI | 69 | 0.9 |
| Demographic variables | Marital status | 108 | 1.4 |
| Demographic variables | Ethnicity | 54 | 0.7 |
| Demographic variables | Admission type | 23 | 0.3 |
| Comorbidities | Myocardial infarction | 39 | 0.5 |
| Comorbidities | Congestive heart failure | 39 | 0.5 |
| Comorbidities | Peripheral vascular disease | 54 | 0.7 |
| Comorbidities | Cerebrovascular disease | 54 | 0.7 |
| Comorbidities | Dementia | 69 | 0.9 |
| Comorbidities | Chronic pulmonary disease | 69 | 0.9 |
| Comorbidities | Rheumatic disease | 85 | 1.1 |
| Comorbidities | Peptic ulcer disease | 85 | 1.1 |
| Comorbidities | Mild liver disease | 108 | 1.4 |
| Comorbidities | Paraplegia | 139 | 1.8 |
| Comorbidities | Renal disease | 162 | 2.1 |
| Laboratory tests | Anion gap, minimum | 85 | 1.1 |
| Laboratory tests | Anion gap, maximum | 85 | 1.1 |
| Laboratory tests | Albumin, minimum | 980 | 12.7 |
| Laboratory tests | Albumin, maximum | 980 | 12.7 |
| Laboratory tests | BUN, minimum | 139 | 1.8 |
| Laboratory tests | BUN, maximum | 139 | 1.8 |
| Laboratory tests | Creatinine, minimum | 139 | 1.8 |
| Laboratory tests | Creatinine, maximum | 139 | 1.8 |
| Laboratory tests | Chloride, minimum | 108 | 1.4 |
| Laboratory tests | Chloride, maximum | 108 | 1.4 |
| Laboratory tests | Glucose, minimum | 193 | 2.5 |
| Laboratory tests | Glucose, maximum | 193 | 2.5 |
| Laboratory tests | Lactate, minimum | 1188 | 15.4 |
| Laboratory tests | Lactate, maximum | 1188 | 15.4 |
| Laboratory tests | Platelet count, minimum | 139 | 1.8 |
| Laboratory tests | Platelet count, maximum | 139 | 1.8 |
| Laboratory tests | Hematocrit, minimum | 162 | 2.1 |
| Laboratory tests | Hematocrit, maximum | 162 | 2.1 |
| Laboratory tests | Hemoglobin, minimum | 139 | 1.8 |
| Laboratory tests | Hemoglobin, maximum | 139 | 1.8 |
| Laboratory tests | Potassium, minimum | 548 | 7.1 |
| Laboratory tests | Potassium, maximum | 548 | 7.1 |
| Laboratory tests | PTT, minimum | 424 | 5.5 |
| Laboratory tests | PTT, maximum | 424 | 5.5 |
| Laboratory tests | INR, minimum | 324 | 4.2 |
| Laboratory tests | INR, maximum | 324 | 4.2 |
| Laboratory tests | PT, minimum | 231 | 3.0 |
| Laboratory tests | PT, maximum | 231 | 3.0 |
| Laboratory tests | Sodium, minimum | 162 | 2.1 |
| Laboratory tests | Sodium, maximum | 162 | 2.1 |
| Laboratory tests | WBC, minimum | 278 | 3.6 |
| Laboratory tests | WBC, maximum | 278 | 3.6 |
| Laboratory tests | Calcium, minimum | 910 | 11.8 |
| Laboratory tests | Calcium, maximum | 910 | 11.8 |
| Laboratory tests | ALT, minimum | 718 | 9.3 |
| Laboratory tests | ALT, maximum | 718 | 9.3 |
| Laboratory tests | ALP, minimum | 779 | 10.1 |
| Laboratory tests | ALP, maximum | 779 | 10.1 |
| Laboratory tests | AST, minimum | 664 | 8.6 |
| Laboratory tests | AST, maximum | 664 | 8.6 |
| Scores | Charlson Comorbidity Index | 849 | 11.0 |
| Scores | SOFA score | 265 | 3.4 |
